# Infertility induced by auxin in PX627 *Caenorhabditis elegans* does not affect mitochondrial functions and aging parameters

**DOI:** 10.18632/aging.103413

**Published:** 2020-06-08

**Authors:** Benjamin Dilberger, Stefan Baumanns, Salome T. Spieth, Uwe Wenzel, Gunter P. Eckert

**Affiliations:** 1 Institute of Nutritional Sciences, Laboratory for Nutrition in Prevention and Therapy, Biomedical Research Center Seltersberg (BFS), Justus Liebig University Giessen, Giessen 35392, Germany; 2 Molecular Nutrition Research, Interdisciplinary Research Center, Justus Liebig University Giessen, Giessen 35392, Germany

**Keywords:** *Caenorhabditis elegans*, mitochondria, FUdR, longevity, PX627, auxin

## Abstract

*Caenorhabditis elegans* is widely used for aging studies. 5-Fluoro-2´-deoxyuridine (FUdR) is commonly used to control offspring. While larvae are stopped from further development, also mitochondrial DNA and function may be affected. Since mitochondria and longevity are closely related, the use of FUdR may falsify possible studies. PX627, an auxin inducible infertility strain to control offspring, allows mitochondrial investigations during senescence without FUdR toxicity.

Longevity and health parameters were assessed in 2- and 10-day old nematodes wild-type N2 and PX627 treated with FUdR or auxin, respectively. Mitochondrial membrane potential, energetic metabolites and reactive oxygen species levels, were determined. mRNA expression levels of key genes involved were quantified using quantitative real-time PCR.

FUdR significantly increased lifespan and health parameters, as well as, mitochondrial function compared to untreated controls and auxin treated PX627. Although a decrease in all parameters could be observed in aged nematodes, this was less severe after FUdR exposure. Glycolysis was significantly up-regulated in aged PX627 compared to N2. Expression levels of *daf-16, sir-2.1, aak-2, skn-1, atp-2 and atfs-1* were regulated accordingly.

Hence, auxin in PX627 might be a good alternative to control progeny, for mitochondrial- and longevity-related investigations in nematodes.

## INTRODUCTION

First introduced in 1974 by Sydney Brenner [1], the free living nematode *Caenorhabditis elegans* (*C. elegans*) represents a well-established model organism, which is commonly used to investigate numerous disorders, ranging from dementia and metabolic imbalances to aging in general. Its relatively small body size, short lifespan and fully sequenced genome, which shows more than 65% homology to humans, are distinct advantages over other animal models [2]. Another inherent advantage is the nematode´s rapid development and ability to reproduce identical offspring as self-fertilizing hermaphrodites. One worm is able to lay approximately 300 eggs after just 3 days [3].

This high number of identical offspring is a problem for nematode-based investigations in general but longevity especially, since larvae cannot be distinguished from their parents once they reach adulthood [4]. This has led to experimental designs focusing on 2-day old adults of the first generation, since larval progeny can be physically distinguished from their parents [5]. This allows the application of external stress to simulate accelerated aging [6]. The use of 5-fluoro-2´-deoxyuridine (FUdR) to chemically sterilize the nematodes, enables investigations in older population without larval contamination [7, 8]. FUdR inhibits DNA synthesis, which does not affect adult nematodes, because all of their somatic cells are post-mitotic, but stops larvae from further development. Consequently, an age-synchronized population without contamination of their progeny, which is essential for lifespan experiments, can be maintained [9]. Mitochondrial DNA (mtDNA), on the other hand, replicates independently of the cell cycle and is affected by FUdR throughout all developmental stages. Consequential alterations in mitochondrial function, due to mtDNA depletion, can be linked to a number of human diseases, such as encephalopathy or myopathy [10]. Furthermore, there is a close relationship between mitochondrial function and longevity [11], making the use of FUdR an unfavourable option for investigations concerned with mitochondrial function, longevity, or their interconnection.

An alternative strategy was first described by Zhang et al., who adapted the auxin-inducible degradation (AID) system into *C. elegans* for a controlled protein depletion [12]. Kasimatis et al. developed the PX627 strain using the AID system to target *spe-44* gene, resulting in a spermatogenesis arrest. Exposure to auxin during the larval stage, therefore, induces infertility, resulting in a progeny-free population without the unwanted effects of FUdR toxicity [13]. Indole-3-acetic acid (auxin) represents a key plant hormone essential for growth regulation and development [14]. Due to its large involvement in regulatory processes of plants, it is commonly used for agricultural purposes [15].

Here we propose PX627 as an ideal system to investigate age-related changes in nematodes and to study the relationship between mitochondrial function and longevity. By comparing PX627 to wild-type N2, we show no differences in life-and health-span, oxidative stress, or mitochondrial function after auxin treatment, which are significantly altered upon FUdR exposure. Especially, mitochondria- and longevity-focused investigations may suffer from this avoidable bias.

## RESULTS

### Lifespan

**Figure 1:**
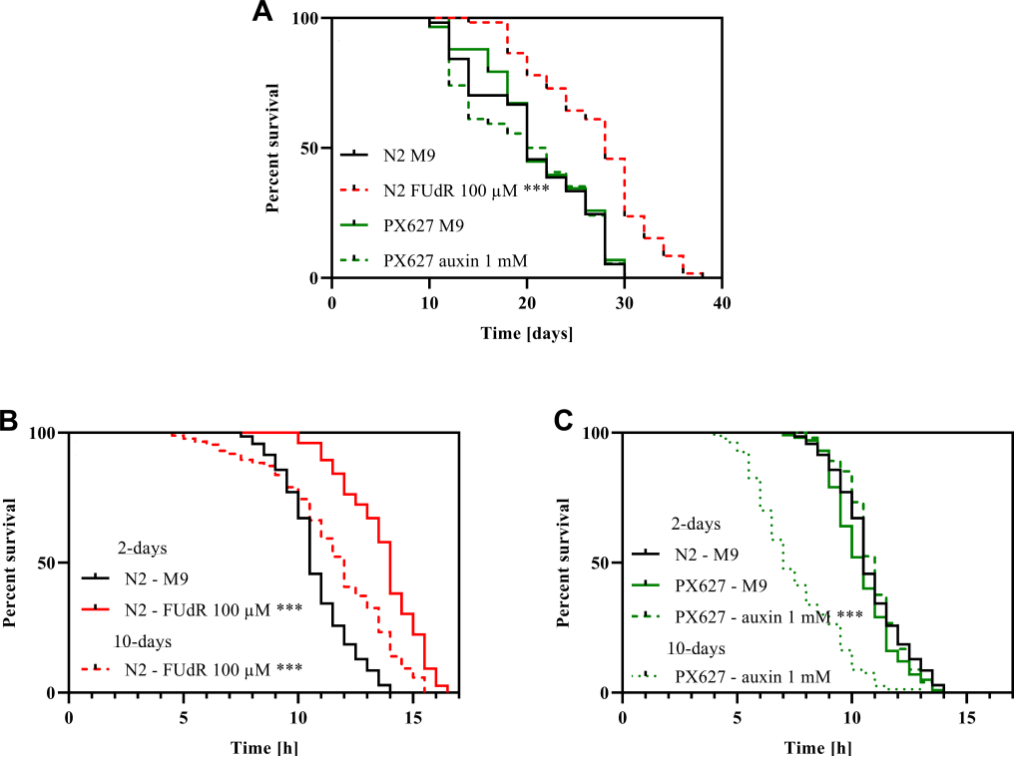
**Survival and stress-resistance:** Survival (**A**) and heat-stress resistance (**B**, **C**) of wild-type N2 and PX627 nematodes unfertilized with either FUdR for N2, or auxin for PX627, at 20°C for physiological and 37°C for stress resistance assessment; n > 54; log-rank (Mantel-cox) test; p*** < 0.001.

The lifespan of wild-type nematodes sterilized with 5-fluoro-2'-deoxyuridine (FUdR) was significantly increased by 40% (p*** < 0.001) after treatment compared to untreated controls. PX627 nematodes, however, showed no alterations compared to wild-type N2, treated with or without auxin (Figure 1A).

### Heat-stress resistance

Chemical sterilization with 100 μM FUdR significantly increased the ability to tolerate heat-stress in 2-day old wild-type nematodes (p*** < 0.001). Although significantly reduced compared to the 2-day assessment (p*** < 0.001), sterilized 10-day old nematodes were still more resilient to heat-stress compared to untreated 2-day old wild-type nematodes (p*** < 0.001) (Figure 1B). With auxin unfertilized PX627 showed no alteration compared to untreated controls and wild-type N2 nematodes at the 2-day assessment, but aged auxin-treated nematodes showed a significantly reduced heat-stress tolerance compared to young controls (p*** < 0.001) (Figure 1C).

### Motility assessment

**Figure 2:**
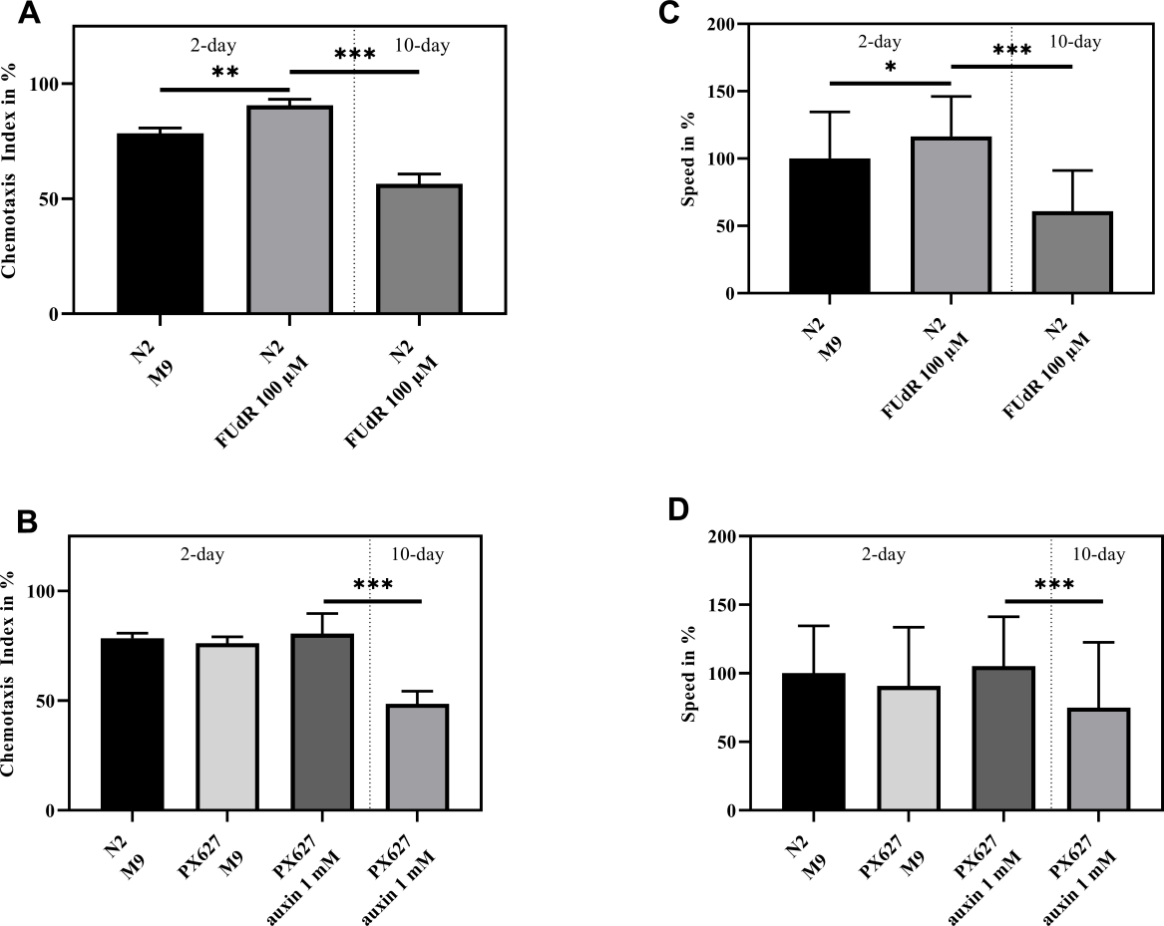
**Health assessment:** Chemotaxis index (**A**, **B**) and Speed (**C**, **D**) of 2- and 10-day old wild-type N2 and PX627 treated and untreated with FUdR or auxin, respectively; n = 6 (Chemotaxis index) and n = 100 (Speed); mean ± standard deviation (SD); one-way ANOVA with Tukey´s multiple post-test; p* < 0.05, p** < 0.01 and p*** < 0.001.

Wild-type N2 moved significantly faster (p* = 0.0236), when sterilized with FUdR, whereas the movement of aged animals significantly slowed down (p*** < 0.001). As for PX627, auxin showed no effects during the 2-day assessment, but again aged nematodes moved significantly slower (p*** < 0.001). Although not significant, it is worth noting, that aged PX627 moved remarkably faster compared to aged N2 (*p* = 0.087) (Figure 2A, 2B).

### Chemotaxis

FUdR significantly increased chemotaxis in 2-day old wild-type N2 (p** = 0.0012). A significant decrease can be observed in 10-day old sterilized nematodes (p*** < 0.001) (Figure 2C). Again, baseline chemotaxis was comparable between untreated N2, untreated PX627 and unfertilized PX627 at day-2, but a significant decrease in the nematodes´ chemotaxis ability could be observed at day-10 (p*** < 0.001) (Figure 2D).

### Mitochondrial membrane potential (ΔΨm)

**Figure 3:**
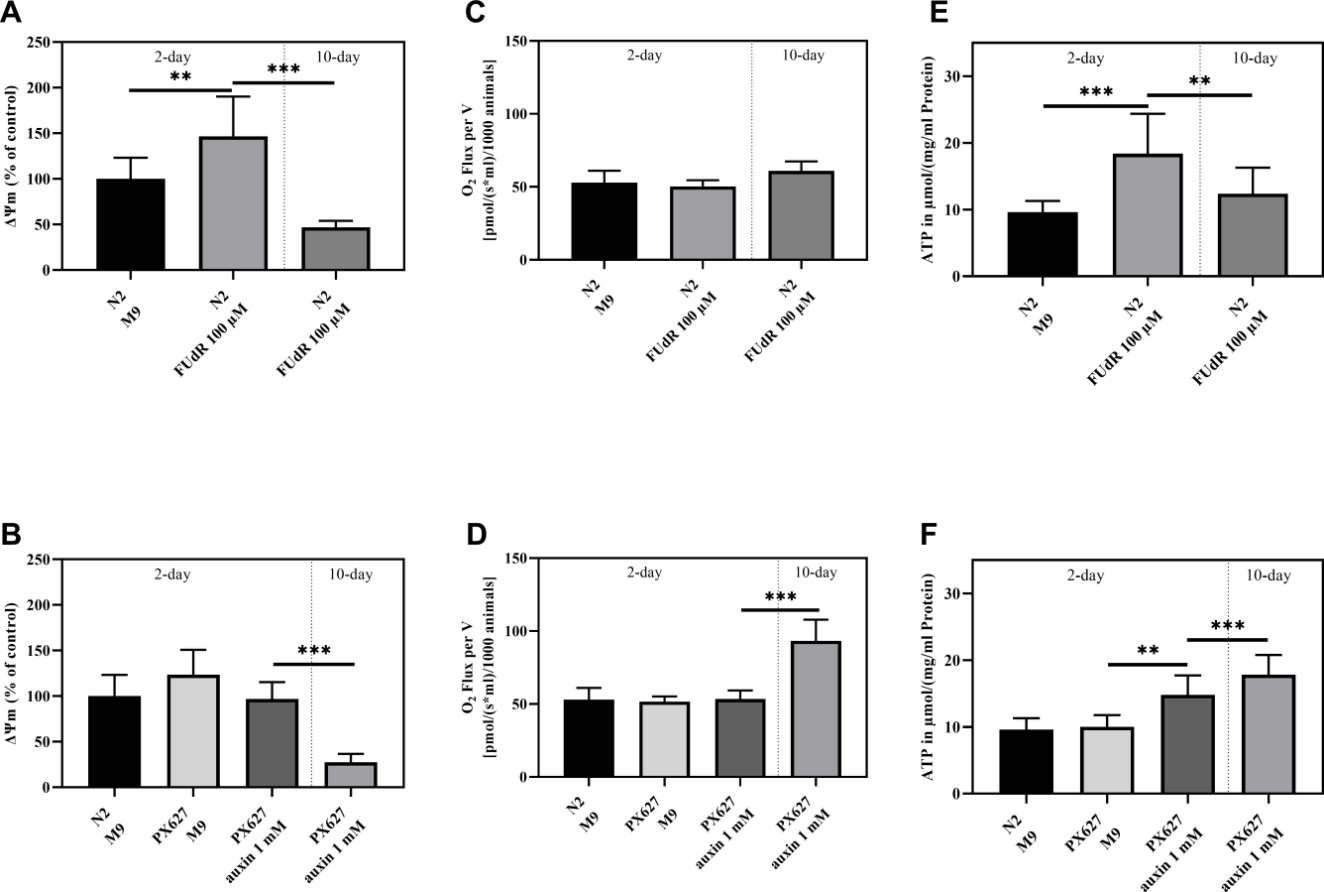
**Mitochondrial function:** Mitochondrial membrane potential (*ΔΨm*) (**A**, **B**), O_2_ Consumption (**C**, **D**) and ATP concentrations (**E**, **F**) of 2- and 10-day old wild-type N2 untreated and sterilized with FUdR and PX627 unfertilized with auxin. For *ΔΨm and ATP* values were normalized to protein concentrations, for O_2_ to the number of nematodes analyzed. n = 8 (O_2_ and *ΔΨm) and n = 7-12 (ATP)*; mean ± SD; one-way ANOVA with Tukey´s multiple post-test; p** < 0.01 and p*** < 0.001.

To evaluate the impact of FUdR and auxin on mitochondrial function, mitochondria were isolated and assessed using Rh123. The ΔΨm was significantly increased upon FUdR exposure in 2-day old N2 compared to the control group (p** = 0.0092), but significantly decreased in old nematodes (p*** < 0.001) (Figure 3A). No alterations could be observed between young wild-type N2 and untreated or auxin-treated PX627, while old PX627 suffered from a significantly decreased ΔΨm (p*** < 0.001) (Figure 3B).

### O_2_ Consumption

Respirational flux was determined using an Oroboros clark-type electrode (O2k Oxygraph, Oroboros Instruments, Innsbruck, Austria). Neither FUdR nor auxin treatment altered the oxygen consumption in the young nematodes of both strains. In contrast, aged PX627, showed a significantly increased O_2_ flux, by approximately 2-fold (p*** < 0.001) (Figure 3C, 3D).

### Adenosine triphosphate (ATP)

As indicated by an improved ΔΨm in young wild-type nematodes, ATP levels were also significantly increased (p*** < 0.001) in sterilized young animals and, again, decreased after senescence (p** = 0.0064). Even though not significant (*p* = 0.5927), the ATP levels of old FUdR-treated N2´s stayed above the baseline of young untreated nematodes (Figure 3E). ATP concentration did not vary between untreated young N2 and PX627 (*p* = 0.9997), whereas auxin treatment caused a significant increase (p** = 0.0071), compared to the ATP concentrations of old PX627 (p = 0.4268) (Figure 3F).

### Mitochondrial structure

**Figure 4:**
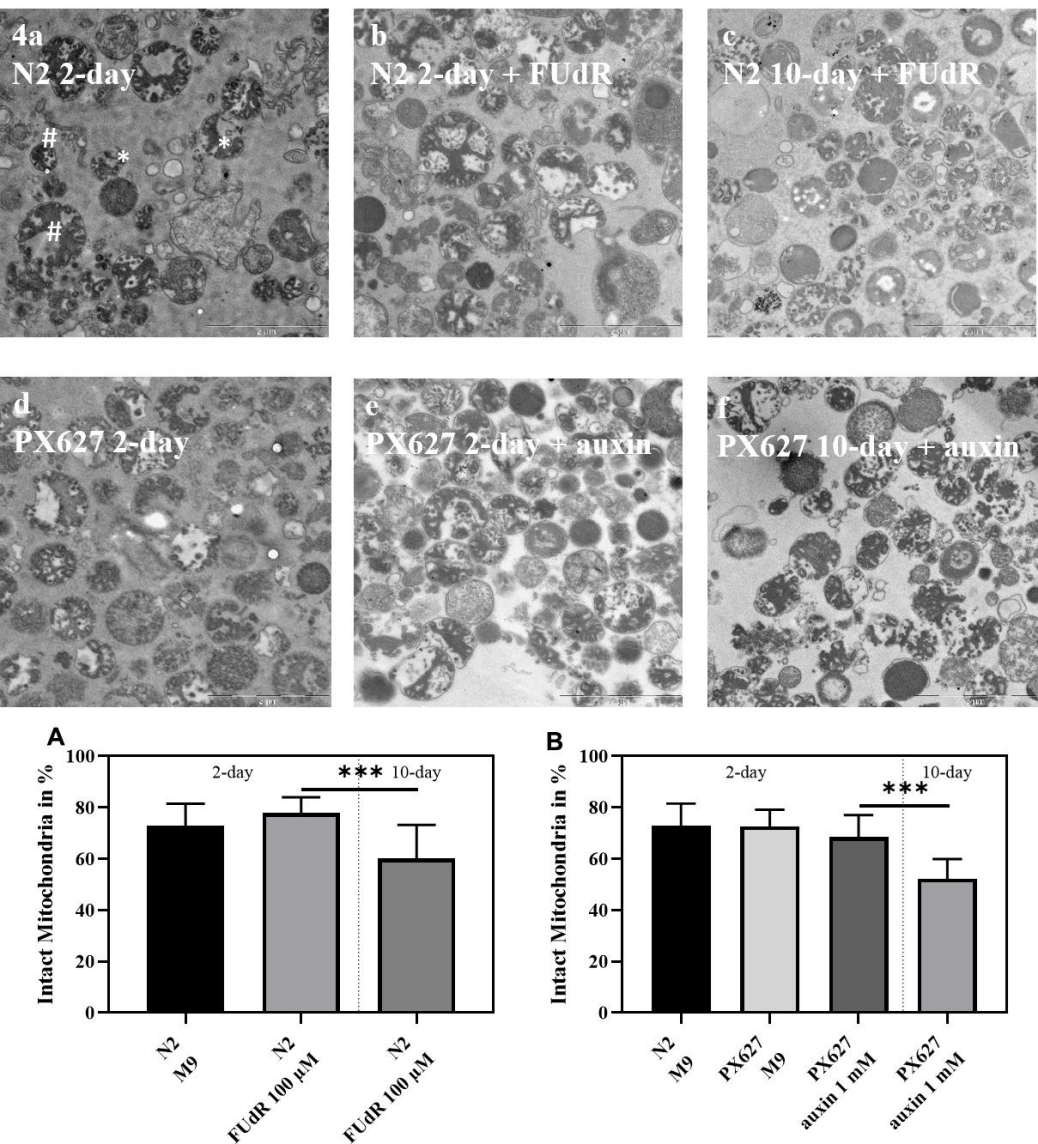
**Mitochondrial structure:** Exemplary electron microscope pictures of isolated mitochondria from wild-type N2 (**a**–**c**) and PX627 (**d**–**f**) nematodes treated with and without FUdR or auxin, respectively. Two examples for intact (#) and fractured (*) mitochondria are indicated in **a**. Scaling bar is 2μM; n = 18-26; mean ± SD; one-way ANOVA with Tukey´s multiple post-test; p*** < 0.001.

To confirm assessed mitochondrial parameters, electron microscopic pictures were taken to evaluate mitochondrial structure. While the young groups of both strains showed no statistical difference, the mitochondrial integrity of wild-type N2 and PX627 nematodes significantly decreased as age progresses (Figure 4; p*** < 0.001). With a mean integrity of 52% the damage was more severe for 10-day old PX627 compared 61% integrity for 10-day old wild-type N2 nematodes. Although not significant, mitochondria of 2-day old FUdR-treated N2 nematodes were, with a mean of 78%, numerically more intact than untreated controls, with a mean of 72% (Figure 4).

### Mitochondrial reactive oxygen species (ROS) measurement

**Figure 5:**
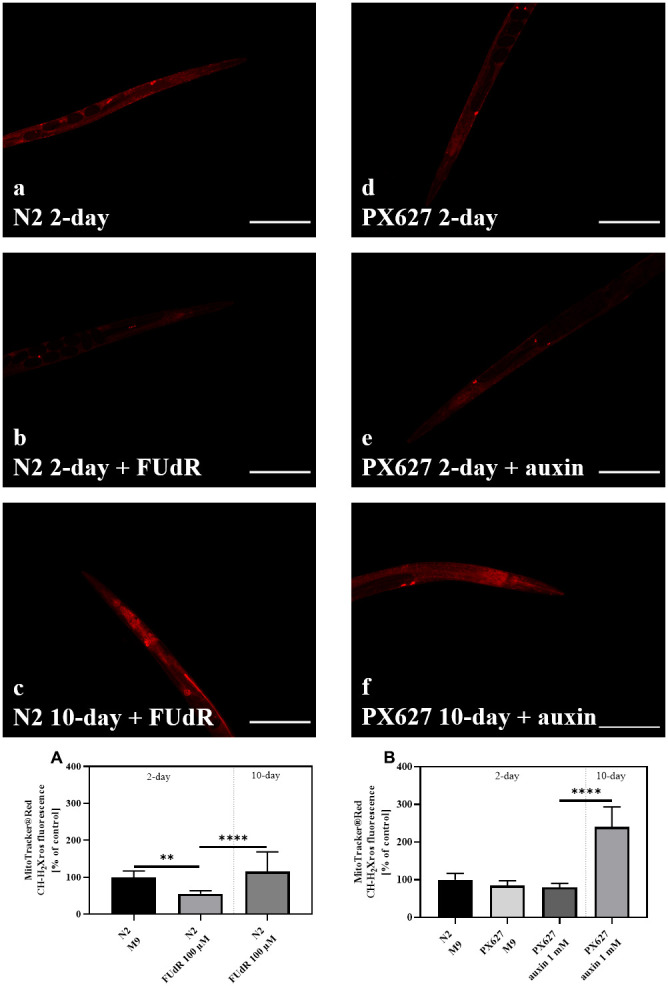
**Reactive oxygen species (ROS) generation: **Wild-type N2 (**a**–**c**) and PX627 (**d**–**f**) nematodes stained with MitoTracker® Red, with and without FUdR or auxin treatment, respectively. Scaling bar is 200μM; n = 20-38; mean ± SD; one-way ANOVA with Tukey´s multiple post-test; p** < 0.01 and p*** < 0.001.

To investigate ROS generation in young and aged nematodes after FUdR or auxin treatment, MitoTracker® Red was applied and mitochondrial ROS-dependent fluorescence measured (Figure 5A–5F).

Two-day old FUdR-treated nematodes showed significantly decreased ROS levels (p** = 0.0011), which significantly increased with age (p*** < 0.001). Compared to untreated young controls, this age-dependent increase was not significant (*p* = 0.6125) (Figure 5A). Young PX627 nematodes, however, showed no differences in ROS, treated or untreated with auxin, compared to wild-type N2. The ROS levels of 10-day old treated PX627 were significantly increased by more than 2-fold compared to all other groups (p*** < 0.001) (Figure 5B).

### Glycolysis

**Figure 6:**
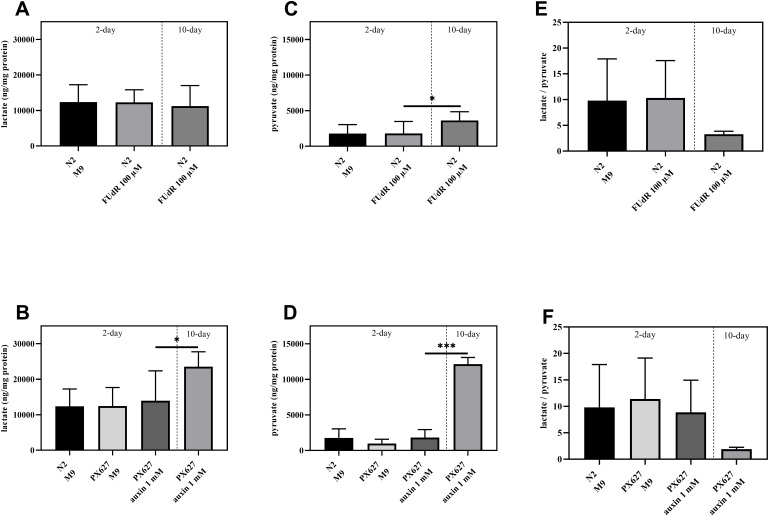
**Glycolytic involvement:** Determination of intracellular lactate (**A**, **B**), pyruvate (**C**, **D**) and lactate/pyruvate ratio (**E**, **F**) of wild-type C. elegans and PX627 treated with 100 μM FUdR or 1 mM auxin, respectively. Values were normalized to protein concentrations. n = 6-15; mean ± SD; one-way ANOVA with Tukey´s multiple post-test; p* < 0.05 and p*** < 0.001.

Glycolytic activity as the second metabolic pathway to provide cellular energy, was assessed, since ATP levels were numerically enhanced in aged N2 and significantly increased in PX627, but ΔΨm, as the driving force for ATPase, was significantly decreased. With the exception of aged PX627 nematodes neither FUdR nor auxin affected lactate or pyruvate concentrations. Old PX627 showed a 5-fold increase in pyruvate compared to 2-day old worms (p*** < 0.001) (Figure 6A–6D). However, the resulting ratio of lactate/pyruvate, as an indicator for glycolysis, was numerically decreased in 10-day old worms (N2: *p* = 0.2004; PX627: *p* = 0.2763), compared to 2-day old treated controls (Figure 6E, 6F), suggesting a lower glycolytic turnover [16].

### Quantitative real-time PCR (qRT-PCR)

FUdR significantly up-regulated the expression levels of longevity-related genes *daf-16* (human forkead box O1 ortholog) in aged and *sir-2.1* (human Sirtuin 1 ortholog) in young N2, as well as *skn-1* (human PGC1α ortholog), a marker for mitochondrial biogenesis, whereas auxin showed no effect in PX627. The expression levels of *aak-2* (human AMP-activated kinase) and *atp-2* (subunit of complex V) were not affected, neither by treatment nor by age. Interestingly, *atfs-1* expression, responsible for mitochondrial unfolded protein response, was significantly decreased in 10-day old auxin-treated PX627, whereas FUdR treated wild-type nematode´s showed a slight, but not significant, upregulation (Table 1).

**Table 1:** Relative normalized mRNA expression levels of 2-day and 10-day wild-type N2 and PX627 nematodes untreated and treated with 100 μM FUdR or 1 mM auxin, respectively.

	**daf-16 *p*-value**	**sir-2.1 *p*-value**	**aak-2 *p*-value**	**skn-1 *p*-value**	**atfs-1 *p*-value**	**atp-2 *p*-value**
**N2 2d M9**	100.0 ± 23.7	100.0 ± 38.0	100.0 ± 68.4	100.0 ± 61.3	100.0 ± 35.8	100.0 ± 16.3
**N2 2d (100 μM) FUdR**	81.0 ± 31.1	243.3 ± 162.1 (p* = 0.023)	73.7 ± 33.3	218.5 ± 105.5 (p* = 0.021)	141.7 ± 47.5	111.0 ± 42.8
**N2 10d (100 μM) FUdR**	126.5 ± 43.3 (*p*+ = 0.022)	171.3 ± 96.7	84.6 ± 48.7	174.3 ± 69.7	137.2 ± 39.5	115.3 ± 33.6
**PX627 2d M9**	96.0 ± 15.3	114.8 ± 89.8	124.1 ± 67.6	122.3 ± 97.7	105.0 ± 23.6	128.7 ± 45.8
**PX627 2d (1 mM) auxin**	90.5 ± 16.3	122.0 ± 62.9	86.2 ± 48.0	96.5 ± 61.3	62.3 ± 14.5	117.8 ± 22.6
**PX627 10d (1 mM) auxin**	78.5 ± 35.2	136.6 ± 67.3	62.0 ± 16.7	98.0 ± 38.8	36.8 ± 10.6	143.6 ± 43.6

mRNA expression of 2-day N2 M9 control is 100%. n = 8; mean ± standard deviation (SD); one-way ANOVA with Tukey´s multiple post-test. * = compared to 2-day N2 M9 control; + = compared to 2-day N2 100 μM FUdR. Results are normalized to mRNA expression of *amanitin.*

## DISCUSSION

The nematode model *Caenorhabditis elegans* is a highly relevant tool for age and genetic related research offering distinct advantages [3]. One drawback, however, is the large number of hermaphroditic progeny, which cannot be separated from their ancestors once they reach adulthood.

The most straightforward strategy, therefore, is to separate the adult animals physically from their offspring before maturation [17]. This approach, however, becomes impracticable once worm populations exceed a critical number impossible to handle. This has led to the development of different techniques to control reproduction. Temperature-sensitive sterile mutant strains, such as fer-15(b26), gon-2(q388), fem-1(hc17), or glp-4(bn2) [18, 19], whose infertility can be induced through a temperature shift from 15°C or 20°C to 25°C, are one strategy. These strains, however, have several drawbacks such as the higher temperature itself, which is no longer physiological for nematodes, and possible unknown interactions caused through genetic manipulation, which have to be taken into consideration [17]. The most commonly applied technique to maintain age-synchronized population, free of progeny, is the use of 5-fluoro-2'-deoxyuridine (FUdR), a DNA synthesis inhibitor, which hinders larvae from further development. Although it does not affect post-mitotic adults, mtDNA and consequently, ATP levels, mitochondrial function and morphology, as well as lifespan can be affected [10]. Auxin exposure, on the other hand, is reported to have no noticeable effects on development, since it is not native to nematodes [12, 13].

Since it is almost impossible to generate large populations of aged nematodes without interference of progeny, we were only able to compare FUdR and auxin treated 10-day old nematodes, instead of untreated controls as well, as conducted for the 2-day assessment. Exemplary pictures of 10-day old nematodes of both strains are supposed to visualize both strategies to control progeny (Supplementary Data). Not only does FUdR stop eggs from further development, but also retains them within their mothers. PX627 nematodes, on the other hand, are able to successfully lay unfertilized eggs. This confirms the findings of Kasimatis et al., reporting an unaltered oogenesis after auxin administration to PX627 nematodes [13]. We therefore argue the inability of FUdR treated nematodes to lay eggs represents a distinct downside on its own, even before taking a closer look at its physiological impact.

The proposed nematode strain PX627 is an ideal model to maintain a synchronous aging population without the adverse effects of FUdR, making it possible to study large populations free of progeny necessary for mitochondrial investigations, with as little confounding and interference as possible. Furthermore, we elucidated age-dependent changes in the mitochondrial function, energy metabolism and expression patterns of key genetic regulators in N2 and PX627 after the application of FUdR and auxin, respectively, shedding light on the high impact of FUdR on mitochondria.

### Life- and health assessment

To study mitochondrial- and longevity-related questions, a nematode strain with a controllable reproductive system, or a substance to induce infertility, should ideally not affect physiological functions such as those of wild-type N2 and should be non-toxic [13]. FUdR, commonly used to sterilize nematodes in concentrations ranging from 50 to 400 μM. Although there are studies reporting no effect of FUdR on the nematodes lifespan [8, 13], the majority of recent publications suggest a significantly increased lifespan of nematodes up to 50% [7, 9, 10, 20], which was confirmed by our own experiments. Even though the physiological impact of FUdR may not be fully understood to this day, here we report a distinct impact on the nematodes physiology. Similar results were observed in a heat-resistance assay. Not only young N2 nematodes treated with FUdR, but also aged ones showed a significantly improved heat-stress resistance compared to 2-day old controls. Improved thermal stress resistance upon FUdR exposure has previously been demonstrated [7, 21], and could be explained by decreased copy number of mtDNA [22]. PX627 nematodes, on the other hand, showed no alterations in lifespan under physiological conditions compared to wild-type N2, neither when treated with auxin nor when left untreated. In addition to our findings under physiological conditions, auxin-induced infertility did not affect 2-day old nematodes´ response to thermal stress, as a marker for aging, in comparison to untreated wild-type N2, whereas 10-day old PX627 suffered from a significantly decreased ability to tolerate heat-stress. To further strengthen these results we investigated the key genetic markers of longevity and stress-resistance, such as *daf-16*, *aak-2* and *sir-2.1* [23–25]. Up-regulation of *daf-16* and *sir-2.1* expression is well documented with longevity and improved stress resistance [26, 27], especially after FUdR exposure [7]. Meanwhile, *daf-16* and *sir-2.1* were mostly unaffected throughout all groups. Aged and young N2 nematodes treated with FUdR, respectively, showed significantly increased mRNA expression levels, which may have contributed to the increased longevity and stress resistance of both groups. Interestingly, however, the expression of *aak-2* slightly, but not significantly decreased in aged auxin-treated PX627. This result could have been expected, since PX627 lack the life-prolonging effect of FUdR and a decreased expression of *aak-2*, as a marker for energy levels, which is in line with increased ATP levels in aged PX627 [28, 29].

Furthermore, the evaluation of the animals´ speed, as a marker of motility, fits with the results of our lifespan and stress resistance assessments. We report the novel findings that young FUdR-treated wild-type worms showed accelerated speed, reflecting a slowed aging process, compared to untreated wild-type worms, as well as to auxin-treated PX627. We observed, however, an age-dependent decrease for both strains.

Only a limited number of studies have investigated the effects of indoles and derivates, such as auxin, on nematodes and findings are somewhat contradictory. While Lee et al. showed a decreased lifespan for concentrations above 0.5 M [30], Sonowal et al. suggested that indoles promote healthy aging in a concentration range of 0.1 – 0.25 mM [31]. Here, we report that 1 mM auxin has no effect on health- and lifespan, which is in line with the findings of Kasimatis et al. who applied the same concentration [13].

The ability to locate a food source offers an alternative aspect of the nematodes´ physiology, focusing on their neurological function and capacity [32, 33]. FUdR treatment significantly improved chemotaxis in 2-day old N2, while aged nematodes suffered from a significantly impaired ability to locate the attractant. Increased chemotaxis is further associated with increased *sir-2.1* expression [27], which is in line with our results.

### Impact on mitochondria and energy metabolism

Although FUdR has no influence on genomic DNA, it interacts with mitochondrial DNA [9, 10, 20]. Thus, we next focused on the impact of FUdR on mitochondrial function, energy metabolism and mitochondria-related oxidative stress. Mitochondria are responsible for the production of ATP as an energetic metabolite through respiration, but also play a key role during aging and the development of several diseases [11]. The mitochondrial membrane potential (ΔΨm) is the driving force for respiration, production of reactive oxygen species (ROS) and ATP synthesis. Thus, it acts as a central parameter for bioenergetics [34], and is reported to decline with aging [35]. While FUdR does in fact decrease the copy numbers of mtDNA, it supposedly has no impact on mitochondrial morphology [10]. Our findings concerning the influence of FUdR on the ΔΨm, however, are novel to the literature. ΔΨm was significantly increased in young wild-type nematodes after FUdR treatment, whereas auxin had no effect in PX627. FUdR may have induced mitogenesis, as indicated by enhanced *skn-1* expression, which could be one explanation for the increased ΔΨm. *Skn-1*, the ortholog of mammalian PGC1α, represents the main driver for mitogenesis in nematodes [36, 37]. Pryzbysz et al. reported that oxidative stress induced by juglone, a reactive naphthochinone, reduces *skn-1* expression in aged nematodes [38].

A close relation between ΔΨm and ROS has been well documented throughout the literature [11, 39]. Our results confirmed that there are no changes between the two parameters, neither with an improved ΔΨm nor with reduced ROS, or vice versa. However, the ROS values are worth mentioning. FUdR treatment increased ROS levels in old compared to young FUdR-treated N2 nematodes, which in turn were significantly decreased compared to untreated young wild-type N2´s. Aged PX627, on the other hand, showed a significant increase of ROS by more than 2-fold compared to all young controls. We argue FUdR masks a physiological ROS generation in aged wild-type´s since it significantly improves the mitochondrial membrane potential. This further strengthens our main hypothesis, that FUdR significantly improves mitochondrial function and mitochondria-associated parameters, thus potentially concealing any beneficial effects of other investigated substances of interest.

ATP, a major energetic metabolite, represents the product of mitochondrial respiration. Rooney et al. reported no effect of FUdR on ATP levels in 4- and 8-day old JK1 mutants nematodes [10]. Yanase et al., on the other hand, showed a significant decrease in wild-type N2 nematodes from day 5 to day 15 after FUdR treatment [16]. However, it is to mention, that not only a different time-point was assessed, but also a different concentration of FUdR applied. We found significantly increased levels in 2-day old wild-type nematodes after FUdR treatment compared to untreated worms, which decreased over time. These findings are in line with the measured ΔΨm data. Interestingly, however, even aged nematodes showed elevated ATP levels after FUdR treatment compared to untreated N2 nematodes. Although energy levels appeared to be elevated in both aged strains compared to their untreated controls, old nematodes moved significantly slower compared to their younger counterparts, suggesting a decreased energy utilization, as previously reported. Van Raamsdonk et al. hypothesized a decreased energy utilization to be responsible for increased ATP levels in *C. elegans* *clk-1* mutants [40]. Thus, it is plausible that ATP may have accumulated in aged worms.

The relation between ΔΨm, ATP and ROS becomes more meaningful when the amount of oxygen consumed by the mitochondria is also taken into account. For wild-type N2´s the oxygen flux stayed the same for all treatment groups, while an improved ΔΨm in young FUdR-treated nematodes accompanied with decreased ROS levels, whereas a decreased ΔΨm resulted in increased ROS levels in aged worms. For PX627, a significantly accelerated oxygen utilization could be measured in aged animals. Again, a decreased ΔΨm in combination with increased ROS was observed, while ATP concentrations were significantly increased. This would suggest increased mitochondrial turnover under suboptimal conditions, requiring increased amounts of pyruvate, as a main substrate for mitochondrial respiration. Especially in aged PX627, but also to a lesser degree in aged N2, severely higher concentrations of pyruvate were measured. Even though not significant a slight up-regulation of *atp-2*, as an essential subunit of complex V [11], could be observed, thus further explaining the ATP increase during senescence. Visualization of mitochondrial integrity by electron microscopy revealed the the intactness of the mitochondrial outer membrane reflects the previously described findings. To the best of our knowledge, this is the first investigation to take a closer look at the interplay between ΔΨm, ROS and ATP in combination with age and the use of FUdR.

Particularly intriguing is also the decrease in *atfs-1* expression of aged PX627, which was not observed in N2. *Atfs-1* activates the mitochondrial unfolded protein response (UPR^mt^) in nematodes, thus regulating mitochondrial turnover and repair upon stress [41]. Age and a decline in UPR^mt^ are closely correlated. Stressors, such as paraquat, or longevity have shown to increase UPR^mt^ [11, 42]. Since FUdR up-regulates *atfs-1* expression, we argue this not only contributes to the increased longevity, but also masks physiological mitochondrial turnover, which can be further evidenced by a more severely decreased ΔΨm of aged PX627 compared to N2.

Glycolysis provides cells with an additional pathway to produce energy [43]. Thus, increased glycolytic turnover in aged nematodes could be an alternative explanation for the elevated ATP levels. However, the ratio between lactate and pyruvate, as an indicator for anaerobic glycolysis [16], was unchanged between strains and treatments, but significantly decreased in aged animals. Since neither FUdR nor auxin affected anaerobic glycolytic activity, it does not appear to be a significant source of ATP.

In summary, these results suggest that caution should be taken when using FUdR for aging studies, as it clearly affects the energetic pathways and consequently alters lifespan and mitochondrial function in *C. elegans*. Since mitochondrial function and longevity are closely related, the use of FUdR adds an experimental bias that may obscure the effects of interventions. PX627 offers a good alternative to avoid the influence of FUdR, allowing studies that are closer to the nematodes unaffected physiology.

## MATERIALS AND METHODS

### Chemicals

The chemicals used were of the highest available purity and standard from Sigma Aldrich (St. Louis, MO, USA) or Merck (Darmstadt, Germany).

### Nematode and bacterial strains

*C. elegans* wild-type strain N2 and transgenic strain PX627 (fxls1 I; spe-44(fx110[spe-44::degron])IV) were ordered from the *Caenorhabditis Genetics Center* (University of Minnesota, MN, US). PX627 represents a modified wild-type strain with an inserted auxin-inducible degradation system targeting the *spe-44* gene mediating sterility, backcrossed five times [13]. Nematodes were maintained on nematode growth medium (NGM) agar plates seeded with *Escherichia coli* (*E. coli*)OP50 at 20°C according to standard protocols [1] and as previously described [5].

### Cultivation and treatment

Synchronous larvae were washed twice in M9 buffer, counted and adjusted to 10 larvae per 10 μl. Nematodes were raised in cell culture flasks (Sarstedt, Nürmbrecht, Germany) or OP50 spread NGM plates. OP50-NGM was given as a standardized food source with a volume 4.4-fold of the larvae containing M9 solution used. L1 larvae were maintained under continuous shaking at 20°C, reaching adulthood within 3 days.

PX627 were treated with 1 mM indole-3-acetic acid (auxin, Alfa Aesar, Haverhill, MA, USA) to induce infertility during the L3 stage [12, 13]. Wild-type N2 nematodes were treated with 100 μM 5-fluoro-2´-deoxyuridine (FUdR) or M9 control after reaching young adulthood. PX627 were also treated with M9 control 48 h prior to the assessment on day-2. The day upon which the nematodes reached young adulthood was defined as day 1. To ensure *ad libitum* food supply until day 10, at day-2 and day-6, old OP50-NGM was discarded after sedimentation of gravid nematodes and fresh OP50-NGM, with effectors incorporated, was given. For the measurements on day-2, FUdR- and auxin-treated young controls and untreated nematodes were co-assessed, whereas at day-10, only sterilized and unfertilized animals could be assessed.

### Lifespan assay

After completing the L4 larval stage, 60 healthy animals per group were transferred onto fresh NGM *E. coli*-containing plates with a sterilized platinum wire. Effectors were incorporated into the OP50 culture with the necessary concentration. To distinguish nematodes of the first generation from their offspring all groups were transferred to new plates every two days, using a platinum wire, until egg-laying of untreated groups stopped. Afterwards nematodes were only transferred onto fresh plates to maintain a sufficient food supply, to reduce stress onto the nematodes during “pick-up”. During separation, nematodes were checked for vital signs using a hot platinum wire held next to the animals´ heads. Worms showing no reaction to the heat stimulus were considered dead. Animals that crawled off the plates, were killed during transfer, or dug themselves into the agar were censored from the experiment. The lifespan curves were statistically compared using the log-rank test.

### Heat-stress resistance assay

To elaborate the nematodes´ resistance to 37°C heat-stress a microplate thermo-tolerance assay was applied. In brief, a nylon mesh (Dr. Fill^®^, Giessen, Germany), if necessary, was used to wash nematodes with M9-Tween® 20 buffer in orderto discard progeny and residual bacteria. Each well of a black 384-well low-volume microtiter plate (Greiner Bio-One, Frickenhausen, Germany) was prefilled with 6.5 μl M9-buffer/Tween® 20 (1% v/v). Subsequently, one nematode was immersed in 1 μl M9 buffer under a stereomicroscope (Breukhoven Microscope Systems, Netherlands). A volume of 7.5 μl SYTOX™ green (final concentration 1 μM; Life Technologies, Karlsruhe, Germany), which penetrates only into cells with compromised plasma membrane and becomes fluorescent after binding to DNA, was added for fluorescence detection. Plates were sealed with a Rotilab sealing film (Greiner Bio-One, Frickenhausen, Germany) to prevent water evaporation. Heat shock (37°C) was applied and fluorescence measured with a ClarioStar Platereader (BMG, Ortenberg, Germany) every 30 min over the course of 17 h. The excitation wavelength was 485 nm and the emission 538 nm.

### Chemotaxis assay

Agar plates were divided into four quadrants. Sodium azid (0.5 M) was mixed in same parts with ethanol (95%) as control, or diacetyl (0.5%) as attractant. A volume of 2 μl for either control or attractant was added to the center of two opposite quadrants. After separation from larvae and residual bacteria, approximately 150 animals were placed in the center of the plates. After 1 h, each quadrant was counted and a chemotaxis index calculated ((number of attractant – number of control) / number total) [32, 33].

### Motility assessment

On the day of analysis, the animals were washed and separated from larvae and residual bacteria as described above. To induce full motility, nematodes were mixed on an orbital shaker at 400 rpm for 1 min, immediately transferred to an unseeded NGM petri dish, and staged for imaging with a 6 MP monochrome camera and a 16 mm FL high-resolution lens. File collection was initiated 1 min after shaking. Speed was measured by a custom software written in Python (Python Software Foundation) called the WF-NTP (Wide Field-of-view Nematode Tracking Platform) [44]. The code initially detects and subtracts the background, considering non-moving objects such as small particles and shadows from the agar plate. After this operation, the remaining labeled regions are identified as individual worms and the positions of such regions are stored for each frame.

### O_2_ consumption

Populations of 1,000 adult nematodes were thoroughly washed, immersed in M9 buffer into the chamber of an Oroboros O2k Oxygraph (O2k Oxygraph, Oroboros Instruments, Innsbruck, Austria), and oxygen flux measured at 20°C. The provided DatLab software (Version 7.0.0.2, Oroboros Instruments, Innsbruck, Austria) was used for analysis.

### Isolation of mitochondria

A population of 5,000 gravid adults were separated from progeny, if necessary, washed with M9-Tween® 20 and transferred into ice-cold isolation buffer (300 mM Sucrose, 5 mM TES, 200 μM EGTA, pH 7.2). Balch homogenization (Isobiotec, Heidelberg, Germany) was applied to generate a sufficient and mitochondria-enriched fraction as previously described [5]. In short, nematodes were passed through the homogenizer´s chamber five times, leaving a 12 μM clearance, using a 1 ml glass syringes (SGE Syringe, Trajan, Australia). The homogenate was centrifuged at 800 g for 5 min at 4°C (Heraeus Fresco 21, Thermo Scientific, Langenselbold, Germany) to sediment debris and larger worm fragments. The mitochondria-containing supernatant was collected and centrifuged at 9,000 g for 10 min at 4°C. The crude mitochondria containing pellet was resuspended in 70 μl swelling buffer (SWB) (0.2 M sucrose, 10 mM MOPS-Tris, 5 mM succinate, 1 mM H_3_PO_4_, 10 μM EGTA, 2 μM rotenone). Aliquots were shock frozen in liquid nitrogen for determination of protein content.

### Mitochondrial membrane potential (ΔΨm)

Mitochondrial membrane potential was determined in 25 μl of in SWB resuspended isolated mitochondria, using fluorescent dye rhodamine 123 (Rh123) in a black 96-well plate with a ClarioStar Platereader (BMG, Ortenberg, Germany). To ensure mitochondrial integrity, ΔΨm was measured for 30 min and after reaching equilibrium and 500 nM FCCP was added to evaluate the ΔΨm-dependent effect on the quenching of Rh123. The results were normalized to protein content.

### Nematode homogenization

A population of 5,000 adult nematodes per group were harvested, thoroughly washed, shock frozen and boiled for 15 min for enzymatic denaturation. Supernatants were collected, after centrifugation at 15,000 g for 10 min. ATP was assessed immediately and spare samples were stored at -80 °C for lactate, pyruvate and protein measurements.

### ATP measurement

ATP was determined using the ATPlite luminescence assay (Perkin Elmer, Waltham, MA, USA). Luminescence was measured in triplicates, according to the manufacturer´s guidelines, using a ClarioStar Platereader (BMG, Ortenberg, Germany). The results were normalized to protein concentrations.

### Transmission electron microscopy

The same volume of fixative (glutaraldehyde (5%) in 0.1 M cacodylate buffer) was added to isolated mitochondria. After 30 minutes incubation at room temperature, probes were centrifuged at 9,000 g for 10 minutes, the supernatant discarded and replaced with fresh fixative (glutaraldehyde (2.5%) in 0.1 M cacodylate buffer). For post-fixation 1% OsO_4_ in 0.1 M cacodylate buffer was applied for 45 minutes at room temperature. Before staining with 1% uranyl acetate over night at 4°C, samples were embedded in low melting temperature gelatine. To dehydrate the gelatine blocks an ethanol series (10-20 minutes each in 30%, 50%, 70%, 80%, 90%, 96%, 99% and 99% over molecular sieve) was conducted on ice followed by propylene oxide. Samples were embedded in Epon and hardened at 60°C for 24 hours. Slices of 80 nm diameter werecut and placed on copper mesh grids, prior to staining with uranyl acetate and lead citrate. For examination, a Leo 912 AB Omega Electron Microscope (Carl Zeiss, Oberkochen, Germany), was used.

For evaluation, pictures were randomized by a third party, to exclude an eventual evaluation bias. Mitochondria were assessed according there structural integrity and divided into two categories: “intact” – cristae structure and outer membrane appears intact or only slightly damaged (**#**); “fractured” – cristae and outer membrane are heavily ruptured or fragmented (*). Categorization was conducted by two independent investigators.

### Mitochondrial ROS measurement

To determine mitochondrial ROS levels, the nematodes were incubated for 48 h with 0.5 μM MitoTracker® Red CM-H2XRos (Fisher Scientific, Schwerte, Germany). MitoTracker® Red accumulates to a high extent at the inner mitochondrial membrane, showing an increased fluorescence upon elevated ROS mainly associated with mitochondria. For epifluorescence microscopy (EVOS FL digital fluorescence microscope, AMG, Bothell, USA), worms were washed with M9-buffer/Tween®20 (1% v/v) solution and anesthetized by addition of 2 mM levamisole. Nematodes were transferred onto a labeled glass slide and covered with a cover slip. The dye was visualized using the EVOS LED Light Cube RFP, with excitation at 531 ± 40 nm and emission at 593 ± 40 nm. Images were taken at a 10-fold magnification. For each group, at least 20 nematodes were photographed. For quantification of fluorescence intensity, only the head region was taken into consideration, using the ImageJ (National Institute of Health (NIH)) software.

### Colorimetric assessment of lactate and pyruvate content

Frozen homogenate samples were slowly thawed to room temperature. Lactate and pyruvate were assessed using two colorimetric assay kits from Sigma Aldrich following the manufacturer´s guidelines (Sigma Aldrich, St. Louis, MO, USA) with a ClarioStar Platereader (BMG, Ortenberg, Germany) and normalized to protein concentrations.

### Protein quantification

Protein concentrations were assessed according to the Pierce™ BCA Protein Assay Kit (Thermo Fisher Scientific, Waltham, MA, USA). Bovine serum albumin was used as a standard.

### Quantitative real-time PCR (qRT-PCR)

Total RNA was isolated using the RNeasy Mini Kit (Qiagen, Hilden, Germany) according to the manufacturer´s guidelines. Complementary DNA was synthesized from 1 μg total RNA using an iScript cDNA Synthesis Kit (Bio-Rad, Munich, Germany). qRT-PCR was conducted using a CfX 96 Connect™ system (Bio-Rad, Munich, Germany) with primers purchased from Biomers (Ulm, Germany). Oligonucleotide primer sequences, primer concentrations and product sizes are listed in Table 2. Gene expression levels were normalized to amanitin resistant (*ama-1*) and actin (*act-2*). For a detailed description see our previous publication [11]. According to the MIQUE guidelines a melting curve was prepared for each primer pair resulting in one significant melting peak.

**Table 2:** Oligonucleotide primer sequences and product sizes for qRT-PCR.

**Primer**	**Sequence**	**Product size (bp)**
*aak-2*	5´-tgcttcaccatatgctctgc-3´	219
	5´-gtggatcatctcccagcaat-3´	
*ama-1*	5´-ccaggaacttcggctcagta-3´	85
	5´-tgtatgatggtgaagctggcg-3´	
*act-2*	5´-cccactcaatccaaaggcta-3´	168
	5´-gggactgtgtgggraacacc-3´	
*atfs-1*	5´-tcggcgatcgatcagctaac-3´	75
	5´-agaatcagttcttggattagggga-3´	
*atp-2*	5´-tccaagtcgctgaggtgttc-3´	151
	5´-aggtggtcgagttctcctga-3´	
*daf-16*	5´-tcctcattcactcccgattc-3´	175
	5´-ccggtgtattcatgaacgtg-3´	
*sir-2.1*	5´-tggctgacgattcgatggat-3´	179
	5´-atgagcagaaatcgcgacac-3´	
*skn-1*	5´-acagggtggaaaaagcaagg-3´	246
	5´-caggccaaacgccaatgac-3´	

The concentration was 0.1 μM for all primers. bp: base pairs.

### Statistics

Unless otherwise stated, values are presented as mean ± standard deviation (SD). Statistical analyses were performed by applying a one-way analysis of variance (ANOVA) with Tukey´s multiple comparison *post-test* (Prism 8.3 GraphPad Software, San Diego, CA, USA). Statistical significance was defined for *p* values *p* * < 0.05, p** < 0.01, and p*** < 0.001.

### Data availability

The dataset generated during this study is available from the corresponding author on reasonable request.

## Supplementary Material

Supplementary Data

Supplementary Figure 1
