# Supplementary material for: Infertility induced by auxin in PX627 Caenorhabditis elegans does not affect mitochondrial functions and aging parameters
Source: Aging (Albany NY). 2020 Jun 8;12(12):12268–84. doi: 10.18632/aging.103413 (PMC7343439; doi:10.18632/aging.103413)
Supplement: Supplementary Data [file aging-12-103413-s001..pdf]

SUPPLEMENTARY DATA

Supplementary 1 egg laying

Two exemplary pictures of aged FUdR-treated N2 and auxin-fed PX627 to show the visible effect of FUdR and auxin on aged nematodes. While the use of FUdR causes a developmental arrest resulting in an inability to lay eggs (Supplementary Figure 1A), auxin leads to the development of unfertilized eggs which can be situated normally (Supplementary Figure 1B).
