# Supplementary material for: Infertility induced by auxin in PX627 Caenorhabditis elegans does not affect mitochondrial functions and aging parameters
Source: Aging (Albany NY). 2020 Jun 8;12(12):12268–84. doi: 10.18632/aging.103413 (PMC7343439; doi:10.18632/aging.103413)
Supplement: Supplementary Figure 1 [file aging-12-103413-s002..pdf]

SUPPLEMENTARY FIGURE

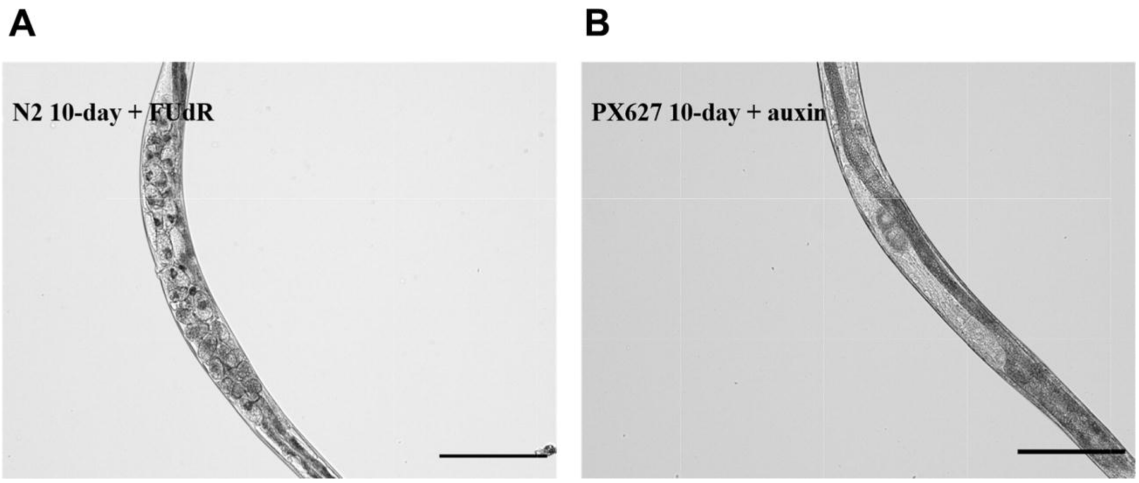

Supplementary Figure 1. Exemplary picture of aged wild-type N2 (**A**) and PX627 (**B**) displaying the inability of N2 to lay their eggs, compared to physiological egg laying of PX627. Scaling bar is 200μM.
